# A novel resident-as-teacher curriculum: the role of experiential learning and coaching

**DOI:** 10.15694/mep.2017.000168

**Published:** 2017-09-22

**Authors:** Amy Tan, Oksana Babenko, Alyssa England, Paul Humphries, Tracey Hillier

**Affiliations:** 1 University of Calgary; 2 University of Alberta

**Keywords:** resident-as-teacher, faculty development, coaching, direct observation, family medicine residents

## Abstract

This article was migrated. The article was marked as recommended.

**Background:** Canadian family medicine residency programs have the challenge of training in a wide breadth of topics and competencies within a two-year program, including training residents to be effective teachers. There has been a gap in knowledge with regards to the most effective method to train residents to teach. We developed, implemented, and evaluated a novel multi-level resident-as-teacher (RAT) coaching curriculum to provide training and authentic experiences for family medicine residents in teaching medical students.

**Methods:** A curriculum centred around multi-level coaching was designed where family medicine faculty members directly observed and provided feedback to family medicine residents teaching small group clinical skills to first and second year medical students. Family medicine residents received didactic training on how to provide effective feedback to students and manage small group dynamics, after reviewing the learning objectives that students were to achieve. This was followed by the authentic small group teaching experiences. A survey was sent out by email to all residents and faculty members who had participated in the RAT curriculum at the end of the 2013-2014 and 2014-2015 academic years. Quantitative survey data were analyzed using descriptive statistics (frequencies, percentages, correlation coefficients (Spearman’s rho)). Qualitative analysis was completed through thematic analysis of respondents’ written comments to open-ended survey questions.

**Results:** 80% of 127 residents strongly agreed (26%) or agreed (54%) that the RAT program effectively developed their teaching skills. 57% either strongly agreed (17%) or agreed (40%) that the direct observation and feedback from faculty coaches helped to improve their teaching skills. There was a significant positive correlation between residents’ perceptions of the usefulness of the feedback from faculty coaches and residents’ perceptions of the overall RAT program’s effectiveness in developing their teaching skills (r=0.42; p=0.001).

Qualitative analysis revealed that residents perceived the RAT program to have solidified their own knowledge base for the content covered in the sessions. Residents also perceived a benefit of near-peer teaching for the medical students and an elevated family physicians’ profile as teachers. They found the active learning experience increased their self-awareness of their teaching skills. Time away from clinical rotations and preparation time were derived as a potential drawback of the program.

All faculty coaches agreed or strongly agreed that the RAT curriculum improved the teaching skills of family medicine residents. Thematic analysis of the faculty coaches’ comments revealed that participating as coaches allowed for their own professional development in that their feedback and coaching skills improved.

**Conclusions:** Our experiences and program evaluation of a novel multi-level resident-as-teacher coaching curriculum show that direct observation with feedback of authentic teaching activities is highly valued, and appears to be effective in developing resident teaching skills while fostering interest in future teaching.

## Introduction

In this era of reduced resident duty hours, it is imperative that residency programs provide high-quality educational teaching and experiences, while ensuring that the delicate balance between residents as learners and health-care providers be optimized ( Chokshi et al., 2017). Residency programs are also required to train residents to teach to achieve accreditation standards. There remains, however, a lack of best practice knowledge on how to train residents to be able to teach junior colleagues (i.e., medical students) effectively (Bree et al., 2014). The combination of these pressures within residency programs means that we need to ensure that residents are provided curricula that satisfy accreditation standards, while making that time away from clinical learning of high value for resident education.

Residents have played an integral part in undergraduate medical education for many years ( Jarvis-Selinger et al., 2011). Accreditation bodies for both undergraduate and postgraduate education programs in Canada require that residents be formally trained to teach medical students ( Jarvis-Selinger et al., 2011). Most resident-as-teacher (RAT) curricular interventions in North America have increased residents’ self-assessed reactions ( Lacasse et al. 2009, Julian et al., 2007) in various residency programs and disciplines through a myriad of teaching modalities. Such modalities have included didactic lectures, small group case discussions, online modules, and simulation exercises in a variety of implementation formats (workshops, intermittent seminars, online modules, flipped classroom) ( Lacasse et al., 2009; Chokshi et al., 2017). While family medicine residents may be well-positioned to aid in the increasing demand for generalist teachers for medical students in Canada, there is even less in the literature about how to train Canadian family medicine residents in teaching skills, given such unique considerations as the shorter residency program length and the wide breadth of the discipline ( Lacasse et al., 2009).

Concurrent with the lack of identified best practices for training family medicine residents to teach, there are also discrepancies as to the best way to assess changes in residents’ teaching skills after such RAT interventions. Kirkpatrick’s (1976) model of evaluation for educational programs outlines four levels of evidence for a program’s outcomes ( Kirkpatrick, 1976; Dannaway et al., 2016). These levels are 1) reactions, 2) learning, 3) behaviour, and 4) results ( Kirkpatrick, 1976). Post and colleagues (2009) recommend Objective Structured Teaching Exercises (OSTE) for assessing residents’ teaching skills. Ostapchuk and colleagues (2010) used clerkship students’ course evaluations and a subsequent focus group to assess residents’ teaching skills following a curricular intervention for a varied group of residents. There are no studies that we are aware of on how to assess residents’ teaching skills within a competency-based medical education framework.

Therefore, we developed, implemented, and evaluated a novel experiential RAT curriculum, that focussed on authentic teaching encounters under faculty guidance, in the competency-based family medicine residency program at the University of Alberta, Canada.

## Methods

We used the concept of coaching, shown in primary and secondary education to help teachers with implementing new teaching behaviours ( Kretlow & Bartholomew, 2010; Wood et al., 2016), to design a coordinated curriculum. This approach aimed to increase the number of generalist teachers for medical students at our university, while explicitly developing the teaching skills of our family medicine residents. Furthermore, we intended to assess changes in our residents’ teaching skills within a competency-based framework by using direct observations during small group teaching sessions and field notes to document coaches’ feedback of residents ( Kretlow & Bartholomew, 2010).

### Description of the RAT curriculum

Using the work of Mann and colleagues (2007) as an overall framework for curriculum development and evaluation, we developed, implemented, and evaluated the overall RAT curriculum. A multi-level curriculum was designed with the needs and objectives of three levels of learners and teachers in mind ( Figure 1). Residents taught first and second year medical students clinical skills (communication and physical examination skills) within the new Physicianship course, in a structured small group format, while being directly observed and coached by family medicine faculty members. Each resident taught four to six small group sessions, each two-hours in duration, over an academic year. Objectives had been developed for the medical student small groups by the Physicianship Course Committee within the medical school (MD Program). The responsibility of covering these objectives for the students resided with the resident teacher. These objectives were reviewed in mandatory training sessions delivered to the residents. The family medicine residency program’s Resident-as-Teacher working group developed the objectives for the residents to achieve as a mandatory part of the residency training program. Finally, this working group also developed a framework to explicitly describe the role of the faculty coach, and ensure clarity of roles for all groups involved.

### Resident training session (prior to small group sessions with medical students)

We reviewed the logistics, flow, and medical students’ objectives of the small groups for communication and physical examination skills with the residents during a didactic session in August, prior to the Fall Term. At this session, residents were also explicitly taught *how* to teach clinical skills to medical students. Several resources for residents to develop their teaching skills were utilized, including the residency program’s internal “Peer Teaching Feedback form”, The Medical Journal of Australia’s “Teaching on the run tips” series ( Lake and Hamdorf, 2004; Vickery and Lake, 2005), and the University of British Columbia’s “Teaching Tips/Reminders for UBC Residents/Fellows ( http://med-fom-fac-dev.sites.olt.ubc.ca/files/2015/03/Teaching-Tips-Reminder-for-UBC-Residents-Fellows.pdf). In addition, one of the residents in the first cohort of the RAT program in 2013 developed a “how to give feedback” video ( https://www.youtube.com/watch?v=KZSmdyYxVYg#action=share) that incorporated the key principles of providing feedback to the medical student; the video was subsequently included as a RAT resource. All the resources were available to residents over the course of the RAT and residents were encouraged to use them as needed. Finally, we explained to residents the concept and role of a faculty coach as someone who directly observed and provided feedback to residents on their teaching of medical students in small groups. Residents were explicitly told that faculty coaches were not responsible for the actual session content or organization, and that this responsibility resided with the resident.

### Faculty coach training sessions (prior to small group sessions with medical students)

Training sessions were held for the faculty coaches to help them develop skills in coaching and direct observation, as opposed to direct teaching in the small groups. This was achieved using the principles of direct observation and feedback employed in childhood education ( Kretlow & Bartholomew, 2010). While the faculty coaches were briefed on the actual session content and objectives that the resident would be teaching in the small groups, the focus of the faculty development sessions was on discussing and developing each others’ coaching skills.

### Small-group sessions for medical students

Family medicine residents were assigned to teach a small group consisting of 4-5 first or second year medical students. In turn, each group of 4 residents was assigned one faculty coach who rotated between small group rooms to observe the resident teaching his/her group of medical students. The faculty coach directly observed the residents as they were teaching, and provided feedback in written form using the residency program’s field note electronic system, and then verbally in a post-session debrief with the residents.

### Program evaluation of the RAT curriculum

Data collection occurred through voluntary electronic surveys sent by email to residents and faculty coaches by the Family Medicine Undergraduate Education Office. Each survey (consisting of both Likert scale questions and open-ended questions with free text responses) was sent out with two subsequent reminder emails. The surveys were sent for the 2013-2014 and 2014-2015 academic years. To ensure anonymity of responses and encourage greater participation in the survey, we did not collect any demographic data. The surveyed residents came from the respective resident cohort of the Family Medicine Residency program, and as such, are considered to be representative of a Canadian urban-based family medicine residency program.

Quantitative survey data points were analyzed using descriptive statistics (frequencies, percentages, correlation coefficients (Spearman’s rho)). Qualitative analysis was completed through thematic analysis of respondents’ written comments to open-ended questions in the survey. Four members of the research team independently coded written comments and then met at several points to reach consensus for emerging themes in an iterative manner (Crabtree and Miller, 1992).

A waiver for ethics approval was obtained from the University of Alberta Health Ethics Research Board as this was deemed to be program evaluation work.

## Results

### Survey of residents

In 2013-2014, 27 out of 63 (44%) resident participants responded to the electronic survey. Similarly, 28 out of 63 residents (44%) submitted responses to the same survey in the 2014-2015 academic year.

Over 90% of responding residents rated their overall experience with the RAT program as very positive (59%) or somewhat positive (35%). 80% of residents strongly agreed (26%) or agreed (54%) that the RAT program effectively developed their teaching skills; with 19% of the respondents being neutral (neither agreed nor disagreed). 90% of residents reported they were very well prepared (38%) or somewhat prepared (52%) for their RAT roles for teaching medical students. Residents found the following RAT training resources to be very helpful or somewhat helpful: email reminders (97%); objectives for medical students (88%); textbook and handouts (83%); objectives for residents (72%); articles (57%); training sessions (59%); and feedback video (29%). Finally, 57% of responding residents either strongly agreed (17%) or agreed (40%) that the direct observation with feedback from faculty coaches was useful in improving their teaching skills. There was a significant positive correlation between residents’ perceptions of the usefulness of the feedback from faculty coaches and residents’ perceptions of the overall RAT program’s effectiveness in developing their teaching skills (r=0.42; p=0.001).

Thematic analysis of the open-ended text responses from residents found that residents perceived the RAT curriculum to have solidified their own knowledge base for the content covered in the sessions. Residents also acknowledged a perceived benefit of the near-peer teaching for the medical students and that they considered themselves as role models for the medical students. Residents reflected that participating in the RAT increased their self-awareness of their teaching skills and elevated the role of family medicine resident physicians as teachers. A theme that this was a large time commitment with regards to time away from clinical rotations and preparation time was derived as a potential drawback of the RAT curriculum.

### Survey of faculty coaches

For the 2013-2014 academic year, 8 out of 12 faculty coaches responded to the survey. Similarly, 8 out of 11 faculty coaches submitted responses for the 2014-2015 academic year. While there was an overlap of some faculty coaches from one year to the next, different coaches did participate in each academic year.

All faculty coaches agreed or strongly agreed that the RAT curriculum improved the teaching skills of family medicine residents. Similarly, all faculty coaches supported the continuation of the RAT curriculum as a mandatory part of the family medicine residency training program.

Thematic analysis of the faculty coaches’ comments revealed that they thought that participating as coaches allowed for their own professional development in that their feedback and coaching skills improved. Faculty coaches also noted that the time commitment and time away from clinical responsibilities were an expected trade-off for both residents and coaches participating in this program. Faculty coaches were concerned as to how beneficial their feedback was in resident skill development.

## Discussion

We developed a novel curriculum to explicitly train Canadian family medicine residents to teach medical students, that is centred around an authentic experiential teaching activity while being directly observed by faculty coaches. The evaluation results from this curricular innovation indicate that residents valued the authentic experience of teaching medical students in core clinical skills.

Our program evaluation confirms a high value curricular intervention that specifically aimed to incorporate principles of competency-based medical education in its training and assessment of residents’ skills. This curriculum focuses on developing skills of a future family medicine educator while creating a vehicle for family medicine to be role-models for medical students.

This curricular intervention was unique to those found in published literature. Specifically, it aimed to develop small-group teaching skills in family medicine residents, rather than teaching in the clinical setting with patients. The intervention itself not only involved a didactic component, but was centred around real-time experiential learning for the residents. Moreover, residents were observed by faculty members while teaching medical students in small groups and were given timely feedback on their teaching behaviours. Although residents found training resources and, to a lesser degree, didactic training sessions helpful, it was the actual experience of teaching that was most valued by the residents for their skill development. The survey results from both resident and faculty coach participants provide evidence for Levels 1 to 3 (reactions, learning, behavior) according to Kirkpatrick’s model of program evaluation ( Kirkpatrick, 1976). Namely, we elicited observation and documentation of resident behaviours by the faculty coaches, in addition to the residents’ perceptions of their own reactions and learning from the RAT program.

A systematic review of resident-as- teacher training by Post and colleagues (2009) indicates that curricular interventions need to be greater than 3 hours long and that intermittent exposure over time may be required. Watchtel and colleagues (2013) found that a two-hour module for emergency medicine residents consisting of adult learning theory, clinical teaching methods, and giving feedback, improved residents’ interest and comfort with teaching. We found that didactic teaching sessions were not as valuable to our residents. Rather, it was the “learning by doing” that allowed residents to self-assess and reflect on their own comfort with the material to be taught to medical students. Teaching small groups of students required residents to try different techniques to manage a small group with different skill levels and learning styles, and impart effective feedback to students in real-time.

Similarly, the actual coaching required to assess and provide feedback to the residents enhanced the coaching skills of faculty members. This in it of itself, proved to be the most valuable part of the coaches’ own professional development. This ties into the College of Family Physicians of Canada’s *Fundamental Teaching Activities (FTA) Framework* ( Walsh et al., 2015) *,* whereby teaching activities are fostered in a practical guide. The notion of developing coaching abilities, while within the framework of the Clinical Preceptor acting as a “Competency Coach” for learners ( Walsh et al., 2015), can be applied in this instance as well. This may speak to the experiential learning activity of teaching small groups that mirrors the provision of patient care in the clinical setting.

Undergraduate medical education continues to evolve from didactic learning to more experiential learning where higher order skills can be applied to develop medical students’ comprehension and retention of the material. The push for more experiential learning increases the demand for small group teachers, be it for clinical skills training, problem-based case learning, clinical reasoning discussions, or case-based exploration of ethical and professional issues. At the same time, medical school classes in Canada have steadily grown in the last decade which, in turn, increases the need for more small group teachers to keep groups appropriate in size for active learning for each student. Furthermore, there has been an upsurge of requests for generalist teachers to be involved at all levels of medical student education in Canada as a direct response to the *Future of Medical Education in Canada*: *A Collective Vision for MD Education* report produced by the Association of Faculties of Medicine of Canada (2010). Developing robust teaching skills in family medicine residents can help enhance their abilities to contribute to the increased generalist teaching demands following graduation.

### Limitations

As with many survey studies, the response rate of 44% from the residents was not ideal. This may have resulted in positive bias. Another limitation is that we qualitatively analyzed text responses to open-ended survey questions. While we did receive rich text responses from the participants, further probing using focus groups or group interviews may have allowed us to further tease out the nuances in how this curricular intervention may have changed resident teaching behaviours.

### Future directions

Future work could include analysis of the direct observation field notes for the type of feedback provided by the faculty coaches to correlate the content of feedback to what residents perceived as the most beneficial. Work in formally utilizing the College of Family Physicians of Canada’s *Fundamental Teaching Activities (FTA) Framework* to further develop the coaching skills of faculty members and to enhance the quality of their feedback to residents may build on this study. Additionally, exploring the long-term impact of the RAT curricular intervention with regards to whether new teachers felt more prepared to teach within the first years of practice would provide more robust information about the level of competency achieved by the RAT participants. Further studies could also look at how this type of experiential learning enhances interest and involvement in teaching over time.

## Conclusion

We developed and successfully implemented a novel multi-level competency-based resident-as-teacher training program for family medicine residents that centres around real-time experience and coaching. This program continues to thrive as a mandatory component of the family medicine residency program while positively contributing to medical student education. Our experiences and program evaluation from the initial two cohorts show that direct observation with feedback of authentic teaching activities is highly valued, and appears to be effective in developing resident teaching skills and fostering interest in future teaching.

## Notes On Contributors

Dr Amy Tan, MD MSc CCFP(PC) FCFP, is an Associate Professor with the Department of Family Medicine at the Cumming School of Medicine, University of Calgary, Calgary, Alberta, Canada.

Dr. Oksana Babenko, PhD, is an Assistant Professor and Medical Education Researcher with the Department of Family Medicine, Faculty of Medicine & Dentistry, University of Alberta, Edmonton, Alberta, Canada.

Dr. Alyssa England, MD CCFP, is a Clinical Lecturer with the Department of Family Medicine, Faculty of Medicine & Dentistry, University of Alberta, Edmonton, Alberta, Canada.

Dr. Paul Humphries, MD CCFP FCFP is a Professor with the Department of Family Medicine, Faculty of Medicine & Dentistry, University of Alberta, Edmonton, Alberta, Canada.

Dr Tracey Hillier, MD, MEd (HSE), CCFP, FRCPC is an Assistant Professor with the Department of Radiology, Faculty of Medicine & Dentistry, University of Alberta, Edmonton, Alberta, Canada.
