# Supplementary material for: A novel resident-as-teacher curriculum: the role of experiential learning and coaching
Source: MedEdPublish (2016). 2017 Sep 22;6:168. [Version 1] doi: 10.15694/mep.2017.000168 (PMC10885298; doi:10.15694/mep.2017.000168)
Supplement: Peer review history

# Reviewer report: Reviewer response for version 1

**DOI:** 10.21956/mep.19352.r28289

**Published:** 2017-10-03

**Authors:** Peter Dieter

**Affiliations:** 1 AMSE

This review has been migrated. The reviewer awarded 4 stars out of 5 It was fun to read the paper and the paper will motivate teachers and residents in their education and training. The training program was good structured and designed and also based on past experiences. The limitations of the study were already mentioned by the authors: low Response rate ant the qualitative analysis of open-ended questions.

Reviewer Expertise:

NA

No decision status is available

# Reviewer report: Reviewer response for version 1

**DOI:** 10.21956/mep.19352.r28290

**Published:** 2017-09-24

**Authors:** Trevor Gibbs

**Affiliations:** 1 AMEE

This review has been migrated. The reviewer awarded 3 stars out of 5 Although I was unsure if the word novel in the title to this paper was actually appropriate, I did enjoy reading it and feels that it adds to our present literature regarding residents as teachers and family medicine education and training. The structure of the training programme was well designed and appropriately based upon past research. However I did feel that more could have been made of the evaluation, specifically in looking at what the students felt regarding this project and moving the evaluation activity to a Kirkpatrick 4 level, or even using another model, such as the Maxwell evaluation scheme

Reviewer Expertise:

NA

No decision status is available
